# Inhibition of EZH2 Causes Retrotransposon Derepression and Immune Activation in Porcine Lung Alveolar Macrophages

**DOI:** 10.3390/ijms24032394

**Published:** 2023-01-25

**Authors:** Liangliang Zhang, Jian Jin, Weiyun Qin, Jing Jiang, Wenbin Bao, Ming-an Sun

**Affiliations:** 1 Institute of Comparative Medicine, College of Veterinary Medicine, Yangzhou University, Yangzhou 225009, China; 2 College of Animal Science and Technology, Yangzhou University, Yangzhou 225009, China; 3 Joint International Research Laboratory of Important Animal Infectious Diseases and Zoonoses of Jiangsu Higher Education Institutions, Yangzhou University, Yangzhou 225009, China; 4 Jiangsu Co-innovation Center for Prevention and Control of Important Animal Infectious Diseases and Zoonosis, Joint International Research Laboratory of Agriculture and Agri-Product Safety of Ministry of Education of China, Yangzhou University, Yangzhou 225009, China

**Keywords:** alveolar macrophage, EZH2, porcine 3D4/21 cell, innate immunity, retrotransposon, endogenous retrovirus

## Abstract

Alveolar macrophages (AMs) form the first defense line against various respiratory pathogens, and their immune response has a profound impact on the outcome of respiratory infection. Enhancer of zeste homolog 2 (EZH2), which catalyzes the trimethylation of H3K27 for epigenetic repression, has gained increasing attention for its immune regulation function, yet its exact function in AMs remains largely obscure. Using porcine 3D4/21 AM cells as a model, we characterized the transcriptomic and epigenomic alterations after the inhibition of EZH2. We found that the inhibition of EZH2 causes transcriptional activation of numerous immune genes and inhibits the subsequent infection by influenza A virus. Interestingly, specific families of transposable elements, particularly endogenous retrovirus elements (ERVs) and LINEs which belong to retrotransposons, also become derepressed. While some of the derepressed ERV families are pig-specific, a few ancestral families are known to be under EZH2-mediated repression in humans. Given that derepression of ERVs can promote innate immune activation through “viral mimicry”, we speculate that ERVs may also contribute to the coinciding immune activation in AMs after the inhibition of EZH2. Overall, this study improves the understanding of the EZH2-related immune regulation in AMs and provides novel insights into the epigenetic regulation of retrotransposons in pigs.

## 1. Introduction

Tissue-resident alveolar macrophages (AMs), which are the most abundant types of innate immune cells in the lung, play crucial roles in the innate immunity and homeostasis of the lung [1,2]. In mammals, AMs are also key players of the first defense line against a variety of respiratory pathogens, such as influenza A virus [3,4], SARS-CoV-2 [5], African swine fever virus [6], porcine reproductive and respiratory syndrome virus [7], and different pathogenic bacteria [8]. Unsurprisingly, pathogens such as SARS-CoV-2 have evolved to evade the immune detection of AMs in the host [9,10]. The immune response of AMs has a profound impact on the outcome of respiratory infection, and it is important to clarify the mechanisms underlying their immune regulation.

The epigenetic regulation of innate immunity has gained increasing attention in recent years [11]. Polycomb group protein (PcG)-mediated epigenetic modifications have been reported to regulate adaptive and innate immunity [12,13,14]. Particularly, enhancer of zeste homolog 2 (EZH2), which is the methyltransferase catalytic subunit of the Polycomb repressive complex 2 (PRC2), is known to catalyze the trimethylation of H3K27 (H3K27me3) for epigenetic repression [15,16] and plays important roles in immune regulation [17]. For example, EZH2 was reported to regulate the polarization of AMs [18] and the inflammatory response of peripheral and tissue-resident macrophages [19,20]. Interestingly, recent studies suggest that inhibition of EZH2/1 can induce an antiviral state in human foreskin fibroblast cells and affect viral infection [21]. Despite these studies, the mechanism underlying the regulation of EZH2 on immune genes in AMs is yet to be understood.

Apart from immune regulation, deeper mechanistic insights about the immune regulation function of EZH2 have been achieved mainly from tumor-related studies [22,23,24]. Currently, multiple highly specific and efficacious inhibitors of EZH2 (e.g., GSK126, GSK343, EPZ5687) have been developed, and several of them are reported to be promising in treating tumors such as lymphoma [25], melanoma [26], mesothelioma [27], small cell lung cancer [28], and prostate cancer [29]. Mechanistically, recent studies demonstrate that specific families of endogenous retroviruses (ERVs, which belong to a class of retrotransposons and make up ~9% of the human genome) are derepressed in tumors after EZH2 inhibition, which results in double-stranded RNA (dsRNA) generation and then triggers pathologic innate immune activation via the stimulator of interferon genes (STING) pathway [28,29]. This ERV-related mechanism, termed “viral mimicry”, also underlies the effect of some other epigenetic drugs (e.g., DNA-demethylating agents) for cancer therapy [30,31]. Further, given the critical contributions of ERVs to innate immunity regulation [32,33], it is highly desirable to determine whether EZH2 also regulates ERVs in AMs and how it contributes to immune regulation.

In this study, we determined the global transcriptomic and epigenomic alterations in AMs after EZH2 inhibition using porcine 3D4/21 AM cells as a model, focusing on the coinciding immune activation and retrotransposon derepression, and the influence on viral infection. Overall, this study improves the understanding of the EZH2-related immune regulation in AMs and provides novel insights into the epigenetic regulation of porcine retrotransposons.

## 2. Results

### 2.1. Genome-Wide Characterization of the Transcriptional Alterations in Porcine AMs after EZH2 Inhibition

**Figure 1:**
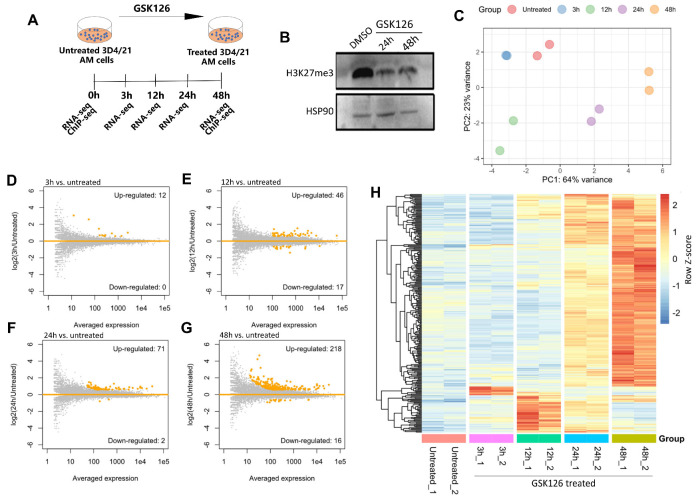
Global transcriptomic alterations in porcine alveolar macrophages after EZH2 inhibition. (**A**) Scheme for the GSK126 treatment of the porcine 3D4/21 AM cells and subsequent experiments. (**B**) Western blot shows the reduction in global H3K27me3 abundance after treatment of the 3D4/21 AM cells with the EZH2 inhibitor GSK126. HSP90 in the bottom panel was used as control. (**C**) PCA plot based on transcriptomic data shows the relationship between different samples. (**D**–**G**) MA plots show the transcriptional alteration after different time periods (i.e., 3h, 12h, 24h, 48h) of GSK126 treatment. (**H**) Heatmap shows the expression pattern of the upregulated genes at different times (i.e., 3h, 12h, 24h, 48h) after GSK126 treatment.

To determine the regulatory function of EZH2 in AMs, we first applied RNA-Seq to determine the genome-wide transcriptional alterations in porcine 3D4/21 AM cells after treatment with the EZH2 inhibitor GSK126 for different time periods (Figure 1A, Table S1). GSK126 is a highly selective EZH2 methyltransferase inhibitor that efficiently inhibits the formation of H3K27me3 [25]. The concentration of GSK126 was optimized as 5 µM by CCK-8 assay (Figure S1), and Western blotting confirmed that the total H3K27me3 abundance was significantly decreased after treatment with GSK126 for 24 h (Figure 1B). Subsequently, RNA-Seq data generated by using the PolyA-enrichment protocol were profiled for the untreated and GSK126-treated samples. All the data generated or integrated in this study are summarized in Table S1.

After confirming the replication of our transcriptomic data by using PCA analysis (Figure 1C), we then performed differential expression analysis to identify the genes with significantly altered transcription after inhibition of EZH2. The numbers of differentially expressed genes (DEGs) range from 12 to 234 (12 at 3 h and 234 at 48 h) after different time periods of GSK126 treatment, with the majority being upregulated and the number of DEGs gradually increasing (Figure 1D–G, Table S2). Further visualization indicates that the DEGs show temporal transcriptional alterations (Figure 1H, Figure S2). For instance, the 12 upregulated genes identified at 3 h were reduced to normal expression soon after (Figure 1H). Furthermore, while the genes induced after 24 and 48 h show substantial overlap, a much higher degree of changes is observed at 48 h (Figure 1H, Figure S2). Together, transcriptomic analyses revealed the temporal activation of hundreds of genes after EZH2 inhibition in porcine 3D4/21 AM cells.

### 2.2. Transcriptional Activation of Hundreds of Immune Genes in Porcine AMs after EZH2 Inhibition

**Figure 2:**
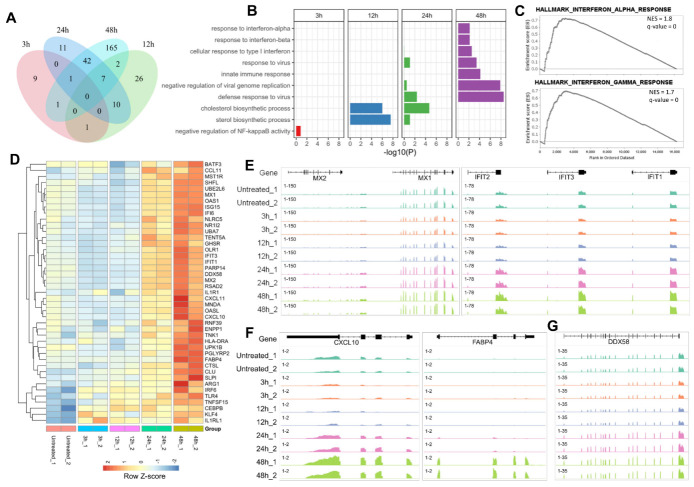
Transcriptional activation of numerous immune-related genes in porcine alveolar macrophages after EZH2 inhibition. (**A**) Venn diagram shows the overlap of the upregulated genes in 3D4 cells after treatment with GSK126 for different time periods. (**B**) GO enrichment for upregulated genes identified at different time points. The top 10 GO terms from the category “Biological Process” were visualized. (**C**) GSEA result for the significantly upregulated genes after treatment with GSK126 for 48 h. Only the top two enriched gene sets were visualized. The normalized enrichment score (NES) and q-values are indicated. (**D**) Expression profile for the immune-related genes that are upregulated after 48 h of treatment. The immune-related genes were selected based on the GO enrichment analysis results. (**E**–**G**) IGV tracks show the expression changes of representative genes related to antiviral effect (*MX1, MX2, IFIT1, IFIT2, IFIT3*), cytokine expression (*CXCL10, FABP4*), and RIG-I pathway (*DDX58*).

After revealing the altered expression of numerous genes after EZH2 inhibition in porcine AMs, we further characterized their expression patterns and functional relevance after different time periods of GSK126 treatment, focusing on the upregulated genes which make up the majority of DEGs. While the upregulated DEGs identified at 24 and 48 h show substantial overlap, those are activated earlier (3 and 12 h) are largely different (Figure 2A). Interestingly, Gene Ontology (GO) analysis suggests that the upregulated genes at later times (particularly at 48 h) are highly associated with immune-related functions (Figure 2B, Table S3), and gene set enrichment analysis (GSEA) further confirmed their association with interferon response (Figure 2C, Table S4). In contrast, genes that are upregulated at 12 h are associated with the sterol biosynthetic process, and those upregulated at 3 h have no significant enrichment (Figure 2B, Table S3). Further visualization confirmed the gradual activation of dozens of immune response genes (Figure 2D-G, S3), including MX1/2 and IFIT1/2/3 which are canonical interferon-stimulated genes (ISGs) with antiviral effects [34] and FABP4 and CXCL10 which are known to regulate cytokine signaling [35]. Interestingly, DDX58 (also known as RIG-I), which is an important receptor for dsRNA sensing [36], is also remarkably upregulated (Figure 2G). Together, these results suggest that numerous immune genes are upregulated after the inhibition of EZH2, which indicates the involvement of EZH2 in the immune regulation in AMs.

### 2.3. IAV Infection Is Remarkably Dampened after Inhibition of EZH2 in Porcine AMs

**Figure 3:**
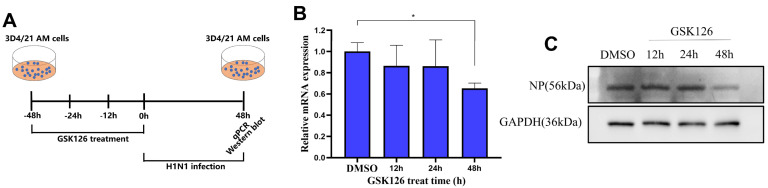
Inhibition of EZH2 remarkably reduces IAV infection in porcine 3D4/21 AM cells. (**A**) Scheme for the H1N1 influenza A virus infection after GSK126 treatment and subsequent experiments on the porcine 3D4/21 AM cells. (**B**) qRT-PCR confirmed the reduced expression abundance of the H1N1 M gene after treatment of the 3D4/21 AM cells with the EZH2 inhibitor GSK126. (**C**) Western blotting confirmed the reduced protein abundance of the H1N1 NP protein after treatment of the 3D4/21 AM cells with the EZH2 inhibitor GSK126. GAPDH in the bottom panel was used as control. Three biological replicates were used for each group. Data are presented as the mean ± SD. p-values were calculated by using Student’s t-test. * *p* < 0.05.

AMs are not only the key players of the first defense line against respiratory pathogens, but also the targets for respiratory pathogen infections [1,6]. Since inhibition of EZH2 promotes the activation of numerous immune genes in 3D4/21 AMs, we further examined how it would affect the infection by influenza A virus (IAV), which is a negative-stranded RNA orthomyxovirus. We treated 3D4/21 cells with GSK126 for different time periods (12, 24, 48 h) and then subjected the treated and untreated cells to H1N1 IAV infection, with all the samples being collected after 48 h of infection for further analysis (Figure 3A). We first performed qRT-PCR to examine the abundance of the M gene of IAV (Table S5) and found that its expression abundance is significantly decreased in the cells pre-treated with GSK126 for 48 h (Figure 3B). Furthermore, Western blotting confirmed that the nucleoprotein (NP) protein abundance of IAV is also remarkably decreased in the cells pre-treated with GSK126 for 48 h (Figure 3C). These results are in line with the activation of immune genes in 3D4/21 AMs after EZH2 inhibition, and they indicate that the antiviral state of AMs can be remarkably enhanced after inhibition of EZH2.

### 2.4. Retrotransposon Derepression Coincides with the Activation of Immune Genes after EZH2 Inhibition

Intrigued by previous studies about the repressive function of EZH2 over transposable elements (TEs) [37] and the links between TEs and innate immune response [32], we further examined how the transcription of TEs is altered after EZH2 inhibition. Initial analysis of the PolyA-enriched RNA-Seq data identified three TE families (LTR4D_SS, LTR6_SS, MER34A1) that are upregulated after 24 and 48 h of EZH2 inhibition (Figure S4). Interestingly, all of them belong to ERVs, and two of them (LTR4D_SS and LTR6_SS) are young ERV families specific to pigs. Given that some ERVs lack the PolyA tail and thus cannot be detected with PolyA-enrichment RNA-Seq [38,39], we generated matched transcriptomic data using the rRNA-depletion protocol for the untreated and GSK126-treated (48 h) 3D4/21 AMs (Table S1). A total of 491 DEGs were identified using the new data (Figure S5), and further analysis confirmed the high similarity of the DEGs identified by using these two types of RNA-Seq data (Figure S6). Therefore, we used the rRNA-depletion RNA-Seq data for an in-depth analysis of TE expression.

**Figure 4:**
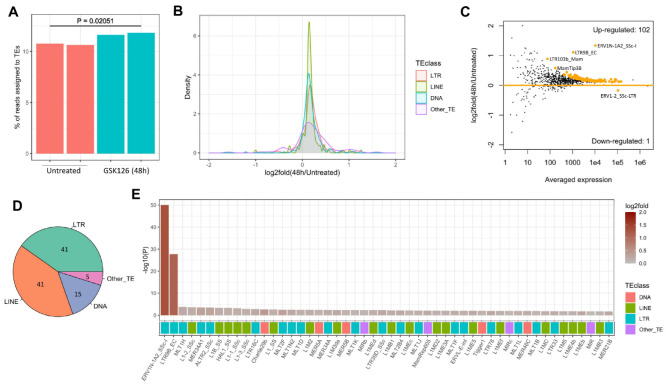
Global derepression of transposable elements in porcine 3D4 cells after EZH2 inhibition. (**A**) Percentages of reads assigned to TEs in 3D4 cells with or without GSK126 treatment (48 h). p-value calculated by using two-sided Student’s test is indicated. (**B**) Density plots compare the altered expression of different classes of TEs, including LTR, LINE, and DNA. (**C**) MA plot shows the altered expression of specific TE families after EZH2 inhibition. TE families with significantly altered expression (P < 0.05) are highlighted in orange color. (**D**) Pie chart shows the proportions of significantly upregulated TE families that belong to each TE class. (**E**) Barplot shows the top 50 significantly upregulated TE families. The color gradient indicates the log2fold expression after EZH2 inhibition (48 h). The *y*-axis indicates the –log10(P). The colored bars at the bottom indicate the memberships of each TE family in the major TE classes, including DNA, LINE, and LTR.

We first examined the numbers of reads assigned to genes and TEs, and we found that the percentages of TE-derived reads are significantly higher in the cells pre-treated with GSK126 (Figure 4A), suggesting the global derepression of TEs due to the inhibition of EZH2 in AMs. Further examination shows that LTRs/ERVs and LINEs (particularly the former) have a higher degree of increased expression relative to DNA transposons and other types of TEs (Figure 4B). Family-level expression analysis identified 102 significantly upregulated TE families; in contrast, only one family is downregulated (Figure 4C, Table S6). Interestingly, the majority (80.2%, 82 out of 102) of the upregulated TE families are LTRs/ERVs and LINEs (Figure 4D,E), both belonging to retrotransposons [40]. The top ten upregulated TE families include five LTR/ERV families (ERV1N-1A2_SSc-I, LTR9B_EC, MLT1H, MER34A1, ALTR2_SSc) and five LINE families (L1-2_SSc, L1B_SS, HAL1_SS, L1-1_Ssc, L1-3_SSc). Manual inspection of the Dfam [41] revealed that many of the significantly derepressed ERV families are young TEs restricted to the porcine lineage (Table S6), yet further knowledge is lacking due to the lack of TE-related studies in pigs. Notably, several of the ancestral TE families (MLT1A, MLT1J, MLT1K, MLT1I) were previously found to be derepressed in human H69 cholangiocytes after inhibition of EZH2 [28]. Overall, these results suggest that after inhibition of EZH2, numerous TE families (particularly retrotransposons) were upregulated, which coincides with the immune activation, and this phenomenon is likely to be conserved at least in some immune and non-immune cells between humans and pigs.

### 2.5. Epigenomic Characterization of the Activated Genes and Retrotransposons after the Inhibition of EZH2

**Figure 5:**
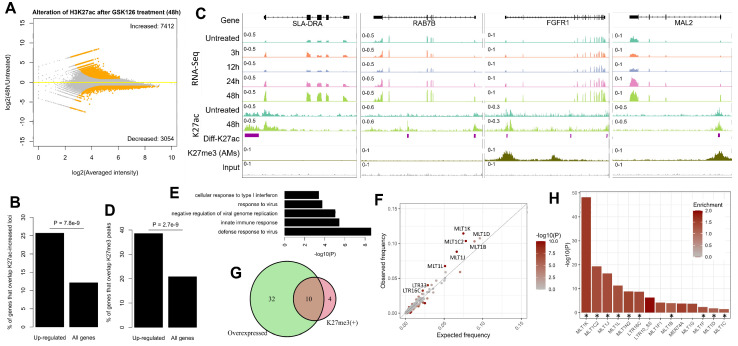
Epigenetic patterns for the activated genes and derepressed retrotransposons after the inhibition of EZH2. (**A**) MA plot shows the altered occupancy of H3K27ac after inhibition of EZH2 in porcine 3D4/21 AMs. The significant differential loci are indicated by orange color. (**B**) Barplot shows that H3K27ac-increased loci are overrepresented near upregulated genes. The two bars represent the percentages of genes (significantly upregulated genes vs. all genes) that overlap the loci with significantly increased H3K27ac intensity. An H3K27ac-increased peak was considered to overlap a gene if it fell within +/- 1 kb of the corresponding transcription start site (TSS). p-value calculated by using binomial test is indicated. (**C**) Integrative Genomics Viewer (IGV) tracks show the differential expression and differential H3K27ac occupancy after inhibition of EZH2 on representative genes, including SLA-DRA, RAB7B, FGFR1, and MAL2. H3K27me3 mark for porcine AMs is also indicated. Loci with significantly altered intensity of H3K27ac (abbreviated as Diff-K27ac) between GSK126-treated (48h) and untreated 3D4/21 AMs are indicated as purple bars in the IGV tracks. (**D**) Barplot shows that the upregulated genes after inhibition of EZH2 are more likely to overlap H3K27me3 peaks. The two bars represent the percentages of genes (significantly upregulated genes vs. all genes) that locate within H3K27me3 domains. A gene was considered to be within an H3K27me3 domain if its TSS overlaps any H3K27me3 peaks. p-value calculated by using binomial test is indicated. (**E**) Top five GO terms enriched within upregulated genes that are out of H3K27me3 domains. (**F**) Scatter plot shows the enrichment of specific ERV families within H3K27me3 domains. (**G**) Venn diagram shows the overlap of the ERV families upregulated after inhibition of EZH2 and overrepresented within H3K27me3 domains. (**H**) ERV families that are significantly enriched within H3K27me3 domains. Those also upregulated after inhibition of EZH2 are indicated by stars at the bottom.

After uncovering the coinciding immune activation and retrotransposon derepression after the inhibition of EZH2, we further characterized their epigenetic patterns, focusing on H3K27ac which marks active regulatory elements [42] and H3K27me3 which is a repressive mark catalyzed by EZH2 [15]. We generated H3K27ac ChIP-Seq data for the untreated and GSK126-treated (48 h) 3D4/21 AMs (Table S1), and comparative analysis identified 10,466 differential binding loci, with the majority (70.8%, n = 7,412) showing increased H3K27ac intensity (Figure 5A). As expected, the transcriptional change is significantly correlated with the alterations of adjacent H3K27ac occupancy (Figure 5B). Notably, we identified a number of genes showing both increased expression and H3K27ac intensity (Figure 5C), including *SLA-DRA* and *RAB7B* which are important for antigen presentation and phagosomes in macrophages [43,44,45]. On the other hand, there are also some upregulated immune genes (e.g., *MX1/2* and *IFIT1/2/3*) lacking increased H3K27ac (Figure S7), likely reflecting the lagged alteration of H3K27ac on many ISGs as previously reported [46]. We further performed TE enrichment analysis and identified seventeen TE families that are significantly enriched within the loci with increased H3K27ac intensity, and most of them are LTRs/ERVs (Figure S8, Table S7). Interestingly, while MER41 elements were previously reported to have facilitated innate immunity evolution in humans [33], the two related pig-specific ERV families (MER41_SS-LTR, MER41B_SS-LTR) were also identified in our study. This suggests that ERVs also played an important role in the immunity evolution of pigs by contributing lineage-specific immune-responsive cis-elements.

To learn more about the mechanisms underlying the immune activation after EZH2 inhibition, the occurrence of the upregulated DEGs within H3K27me3 domains was further examined. We did not have publication-quality H3K27me3 ChIP-Seq data for 3D4/21 AMs; thus, we turned to integrating a recently published dataset for primary porcine AMs [47]. The 3D4/21 cell line is assumed to well match primary AMs since it was originally derived from primary AMs, and as expected, transcriptomic comparison based on newly generated and public data [47,48] confirmed their similarity (Figure S9). We found that 38.5% of the upregulated DEGs (e.g., *BICD1*, *BDKRB2*, *FGFR1*, and *MAL2*) are within H3K27me3 domains, which is significantly higher than expected (*P* = 2.7 × 10^−9^, Figure 5C,D), and these genes are probably under the EZH2/H3K27me3-mediated regulation. On the other hand, DEGs such as *SLA-DRA* and *RAB7B* are unlikely to be directly regulated by EZH2 since they are out of H3K27me3 domains (Figure 5C, Figure S5). Correspondingly, GO analysis shows that only the upregulated genes outside of H3K27me3 domains are significantly associated with innate immune response, yet those in H3K27me3 domains have no enriched GO terms (Figure 5E), suggesting that the immune genes upregulated after EZH2 inhibition are likely to be under indirect regulation of EZH2/H3K27me3-mediated regulation.

Multiple studies suggest that the derepression of ERVs contributes to the innate immune activation in tumors after epigenetic therapy through a “viral mimicry” mechanism that involves dsRNA generation and STING pathway activation [28,29,49]. Given the evident retrotransposon derepression after EZH2 inhibition in porcine AMs (Figure 4), we further examined their overlap with H3K27me3 domains. In total, we identified 14 ERV families that are significantly enriched within H3K27me3 domains (Figure 5F). Among them, ten families also show upregulated expression based on our rRNA-depletion RNA-Seq data (Figure 4E, Figure 5G,H, Table S6); thus, these ten ERV families are most likely under EZH2/H3K27me3-mediated repression. Interestingly, nine of these ten ERV families belong to the ERVL-MaLR superfamily—including two (MLT1J, MLT1K) previously validated to be derepressed in human H69 cholangiocytes after inhibition of EZH2 [28]. Taken together, these results indicate that, either by generating dsRNA or by creating immune-responsive cis-elements, ERVs may contribute to the immune activation in AMs after the inhibition of EZH2.

## 3. Discussion

The epigenetic regulation of innate immunity has received increasing attention in recent years [11]. The core epigenetic regulator EZH2 catalyzes H3K27me3 to achieve PcG-mediated epigenetic repression [15,16], and it also plays critical roles in immune regulation [17]. This study aimed at characterizing the function of EZH2 in AMs, which form the first defense line against various respiratory pathogens and have profound importance in the outcome of respiratory infection. By using porcine 3D4/21 alveolar macrophages as a model, this study comprehensively characterized the transcriptomic and epigenomic alterations after inhibition of EZH2, focusing on the coinciding activation of immune genes and derepression of retrotransposons, and the underlying epigenetic mechanism.

Our study demonstrates that the inhibition of EZH2 causes the transcriptional activation of hundreds of innate immune genes in porcine AMs, including many canonical ISGs such as *MX1/2* and *IFIT1/2/3*. Given that interferon response is important for the inhibition of viral infection [50,51], we further revealed that IAV infection is remarkably suppressed in the porcine AMs pre-treated with EZH2, which suggests the enhanced antiviral state in the porcine AMs after inhibition of EZH2. Interestingly, one previous study showed that inhibition of EZH2 in human foreskin fibroblast cells also caused increased ISG expression and enhanced antiviral state, with the infective potential of several viruses (e.g., herpes simplex virus, Zika virus, cytomegalovirus, and adenovirus) also being remarkably suppressed [21]. Further, given that the inhibition of EZH2 causes innate immune activation in several tumors [28,29], it suggests that the regulation of the innate immune response by EZH2 occurs at least in many different cell types in mammals. Surprisingly, our previous study on Drosophila brain discs showed that despite the global reduction in H3K27me3 levels after the knockout of the PcG recruiters *Pho* and *Spps*, many immune genes were downregulated [15] rather than activated. However, the mechanism underlying this difference observed between Drosophila brain discs and porcine AMs remains unclear. Overall, current knowledge about the function of EZH2 on immune regulation is mainly derived from studies on model organisms (particularly humans and mice), and it remains to be clarified whether such a mode of regulation is prevalent in other cell types from other species.

In addition to the activation of numerous immune genes, we found that specific families of retrotransposons also are derepressed after inhibition of EZH2 in porcine AMs. Most retrotransposons are usually under epigenetic repression [52,53], yet some of them, particularly those belonging to ERVs, can be derepressed during early development [42,54,55] or innate immune response [33,56]. Recent studies suggest that inhibition of EZH2 in tumors causes ERV derepression followed by dsRNA generation, which can then be sensed by the STING/MDA5 pathway and trigger the immune response [28,29]. This “viral mimicry” mechanism also underlies other epigenetic drugs (e.g., DNA-demethylating agents) for cancer therapy [30,31,49]. The derepressed ERV families in our study include both those of ancient origin and those newly evolved in pigs. The pig-specific ERV families remain poorly understood, but, a previous study suggests that several of the ancient families (MLT1A, MLT1J, MLT1K, MLT1I) observed to be derepressed in our analysis are also derepressed in human H69 cholangiocytes after EZH2 inhibition [28]. We assume at least some of the derepressed ERV families are likely under the EZH2-mediated epigenetic repression, and such regulation is likely to be conserved between humans and pigs.

Apart from triggering the innate immune response via “viral mimicry” [49], ERVs are also known to regulate innate immunity by creating cis-elements that are bound and activated by core immune transcription factors such as STAT1 and IRF1 [32]. Multiple studies suggest that ERVs facilitated the lineage-specific evolution of innate immunity in primates [33,55], ruminants [57], and bats [58] by creating interferon-stimulated enhancers, yet similar studies in pigs are still lacking. Even though this study did not focus on interferon-stimulated enhancers, we did identify thousands of epigenetically annotated cis-elements (based on H3K27ac marks) that were activated after the inhibition of EZH2, which provides an opportunity to examine the links between ERVs and immune-related cis-elements in pigs since these cis-elements are likely to underlie the observed immune activation. Interestingly, seventeen TE families are significantly enriched within the activated cis-elements, with the majority belonging to ERVs. Matching previous findings that MER41 and MER41-like (e.g., MER41_BT for cattle) ERVs facilitated the lineage-specific innate immunity evolution in primates and ruminants [33,55,57], we found that two MER41-like ERV families specific to pigs (MER41_SS-LTR and MER41B_SS-LTR) also significantly overlap the cis-elements that were activated after the inhibition of EZH2. These seventeen TE families, including the two MER41-like families, other ERVs (e.g., MLT1K, LTR39B3_SSc, LTR33, LTR39A2_SSc), SINEs (e.g., MIR3, MIRb, MIRc), and LINEs (e.g., L2b, L2c, L2d), may also contribute to pig-specific innate immunity evolution. We expect that an in-depth understanding of the contribution of ERVs to lineage-specific innate immune evolution in pigs will be achieved by further studies involving interferon-stimulation experiments and CRISPR engineering of ERV-derived enhancers.

While our major finding is the regulation of immune genes and retrotransposons by EZH2 in porcine AMs, there are still several limitations to the in-depth understanding of the underlying mechanism. First, our study about the function of EZH2 is largely based on the use of the selective inhibitor GSK126. While the high selectivity of GSK126 has already been well confirmed and extensive studies in the tumor field suggest that the effect of GSK126 in treating tumors is through its inhibition of EZH2 [25,29,49,59], additional EZH2 knockdown experiment may provide further evidence about the function of EZH2. Second, as our study identified fourteen ERV families that are significantly enriched within H3K27me3 domains, including ten families showing upregulated expression after inhibition of EZH2, it would be interesting to determine which of these ERV families show decreased H3K27me3 levels by comparing matched H3K27me3 ChIP-Seq data between untreated and GSK126-treated AMs. We expect the mechanistic links between retrotransposon derepression and immune activation in AMs and probably other cell types will be further investigated by future studies.

In summary, this study uncovered the coinciding retrotransposon derepression and immune activation in porcine AMs after the inhibition of EZH2 and revealed their mechanistic links through comprehensive transcriptomic and epigenomic analysis. We expect this study will improve the mechanistic understanding of the EZH2-dependent immune regulation in AMs and provide novel insights into the epigenetic regulation of porcine retrotransposons.

## 4. Materials and Methods

### 4.1. Cell Culture and EZH2 Inhibitor Treatment

Porcine 3D4/21 alveolar macrophages were purchased from ATCC and cultured with RPMI-1640 medium (SH30809.01, Cytiva, USA) supplemented with 10% FBS (Sigma-Aldrich, USA) and 1% anti-anti (15240062, Gibco, USA) in a constant-temperature incubator at 37 °C with 5% CO_2_ and saturated humidity. When 3D4/21 cells reached about 50–60% confluency, the EZH2 inhibitor GSK126 (S7061, Selleck chem, USA) was added at desired time points (e.g., 3, 12, 24, 48 h before sample collection) to the medium to a concentration of 5 μmol to begin treatment. The same volume of DMSO (21985023, Gibco, USA) was added 48 h before sample collection as the untreated control. After the desired time of treatment, all samples were collected for subsequent experiments.

### 4.2. CCK-8 Assay

The 3D4/21 cells were seeded into a 96-well cell culture plate. When they reached about 60% confluency, the EZH2 inhibitor GSK126 was added to the medium with different concentrations (i.e., 5, 10, 20, 30, 40 μmol), and the samples were collected after 24 h of treatment for the CCK-8 assay. The same volume of DMSO was used as the untreated control. The CCK-8 assay was performed according to the instructions provided with the Cell Counting Kit-8 kit (CK04, Dojindo, Japan).

### 4.3. Influenza A Virus Infection

Porcine 3D4/21 cells were seeded into 12-well cell culture plates and infected with the H1N1 IAV by using 200 µL virus solution per well. The virus inoculum was discarded after adsorption at 37 °C for 2 h, and RPMI-1640 complete culture medium was added to continue the culture. At 12, 24, 36, and 48 h of H1N1 IAV infection, the cytopathic conditions were observed under a microscope, and the cells were collected at 48 h.

### 4.4. Total RNA Extraction, cDNA Synthesis, and qRT-PCR

Total RNA was extracted using the FastPure Cell/Tissue Total RNA Isolation Kit (RC101-01, Vazyme, China) with on-column DNA digestion. The reverse transcription was performed using a reverse transcription kit (R333-01, Vazyme, China), with the reaction system containing 4 µL of 5 × qRT SuperMix II, 1 µg of total RNA, and RNase-free ddH_2_O to a final volume of 20 µL. The reaction program was set as follows: 15 min at 50 °C, 5 s at 85 °C, and then storage at 4 °C. The primers for qRT-PCR were designed using Primer Premier 5.0 and are summarized in Table S5. GAPDH was used as control. All qPCR reactions were conducted in a 10 µL reaction volume with 1 µL of cDNA, 0.2 µL of each primer (10 µmol/L), 5 µL of 2 × AceQ Universal SYBR qPCR Master Mix, and 3.6 µL of ddH_2_O. Thermocycler settings were as follows: 95 °C for 5 min, 40 cycles of 95 °C for 10 s, and 60 °C for 30 s. Three replicates were conducted for all analyses.

### 4.5. Western Blotting

The collected cells were washed with pre-cooled PBS, lysed with a mixture of RIPA lysis buffer and protease inhibitor cocktail (HY-K0010, MedChemExpress, USA) for 10 min at room temperature, and then centrifuged at 10,000 rpm for 10 min at 4 °C. Denaturing was performed with 5 × SDS–PAGE loading buffer at 100 °C for 5 min. After SDS–PAGE, the samples were transferred to a PVDF membrane (Millipore, ISED00010). Blocking was performed with 5% skimmed milk for 2 h at room temperature, and the blocked membranes were incubated with primary antibodies (H3K27me3, 17-622, Millipore, USA; NP, ab104870, Abcam, USA; HSP90, 60318-1-Ig, Proteintech Group, USA; GAPDH, bsm-33033M, Bioss, UK) overnight at 4 °C. After the membranes were washed with PBST, they were incubated with a secondary antibody (SA00001-1, Proteintech Group, USA) for 1 h at room temperature, washed with PBST again, and then exposed on the chemiluminescence imager.

### 4.6. RNA-Seq

Total RNA was extracted by using the FastPure Cell/Tissue Total RNA Isolation Kit (RC101-01, Vazyme, China) with on-column DNA digestion and then submitted to BGI for library construction and sequencing. The two types of RNA-Seq libraries were generated by using the PolyA-enrichment and rRNA-depletion protocols, respectively. The constructed RNA-Seq libraries were sequenced as 150 bp paired-end reads with the BGI MGISEQ-2000 platform.

Raw reads were trimmed with default settings with Trim Galore v0.6.4 (https://github.com/FelixKrueger/TrimGalore accessed on 25 December 2022). Transcripts per million (TPM) values were calculated with RSEM v1.3.2 [60]. To perform differential expression analysis, we aligned trimmed reads to the reference genome (Sscrofa11) using STAR v2.7.3 [61] and then obtained gene-level read counts using the *featureCount* function from subread v2.0.0 [62]. At last, differentially expressed genes were identified using DESeq2 v1.30.1 [63] with the following cutoff: FDR < 0.05 and fold change > 1.5.

### 4.7. ChIP-Seq

ChIP-Seq was performed following our previous study [42]. Chromatin fragmentation was performed using Covaris M220. The amount of chromatin was 20 μg per reaction. The amount of the antibody of H3K27ac (ab4729, Abcam, USA) used was 5 μg per reaction. The obtained ChIP-DNA was submitted to BGI for DNA library construction and then sequenced as 50 bp paired-end reads with the BGI MGISEQ-2000 platform.

Reads were trimmed with TrimGalore v0.6.4 and then aligned to the corresponding reference genome (Sscrofa11) using Bowtie v2.3.5 [64] with default settings. PCR duplicates were removed using the *rmdup* function of samtools v1.13 [65]. After confirming the data reproducibility, reads from biological replicates were pooled together for further analysis. Peak calling was performed with MACS v2.2.6 [66]. Differential binding analysis was performed using DiffBind v3.4.11 [67] with the following settings: minOverlap = 1, summits = 400, method = DBA_EDGER.

### 4.8. Reference Genome and Annotation

The reference genome and gene annotation for pigs (Sscrofa11, release 108) were downloaded from the ENSEMBL database [68]. Transposable element annotations of the corresponding reference genome were downloaded from UCSC genome browsers [69]. Information for specific TE families was manually checked on the Dfam database [41].

### 4.9. Gene Ontology and Gene Set Enrichment Analysis

Gene ontology enrichment analyses for differentially expressed genes were performed using DAVID [70]. Gene set enrichment analysis was performed by using GSEA v4.3.2 [71], with the following settings: Gene sets database = h.all.v2022.1.Hs, Permutation type = gene_set, Metric for ranking genes = log2_Ratio_of_Classes. The gene expression matrix containing the calculated TPM values was used for GSEA analysis.

### 4.10. Transposable Element Analysis

Family-level expression analysis of transposable elements was performed using TEtranscript v2.2.1 [72]. In brief, the reads were first aligned to the reference genome by using STAR v2.7.3 [61] with the recommended settings: --winAnchorMultimapNmax 100 --outFilterMismatchNmax 10 parameters. Subsequently, we used the *TEcount* function to determine the read counts for each gene and TE family, and we used the *TEtranscript* function to determine the TE families with significantly altered expression between the compared samples with the cutoff of FDR<0.05. To determine if certain TE families are overrepresented within given genomic regions (e.g., the peaks for H3K27ac and H3K27me3), we adopted the *fisher* function of BEDtools v2.29.2 [73], which determines the enrichment fold and p-value between two lists of genomic intervals by using Fisher’s exact test.

### 4.11. Statistical Analysis and Data Visualization

All statistical analyses were performed with R statistical programming language [74]. Heatmaps for ChIP-Seq data were generated using DeepTools v3.5.1 [75]. Heatmaps from gene expression clustering analysis were generated during 2022 using pheatmap (https://github.com/raivokolde/pheatmap accessed on 25 December 2022). Representative tracks for RNA-Seq and ChIP-Seq data were visualized using IGV v2.11.1 [76]; the TDF files were generated by using the *count* function of igvtools, and the library size of each sample was normalized to 1 million for visualization.

## Data Availability

All the raw and processed sequencing data generated in this study have been submitted to the NCBI Gene Expression Omnibus (GEO; https://www.ncbi.nlm.nih.gov/geo/ accessed on 25 December 2022) under accession number GSE221479.

## Supplementary Materials

The following supporting information can be downloaded at: https://www.mdpi.com/article/10.3390/ijms24032394/s1.
