# Supplementary material for: Inhibition of EZH2 Causes Retrotransposon Derepression and Immune Activation in Porcine Lung Alveolar Macrophages
Source: Int J Mol Sci. 2023 Jan 25;24(3):2394. doi: 10.3390/ijms24032394 (PMC9917017; doi:10.3390/ijms24032394)
Supplement: Supplementary file 1 [file ijms-24-02394-s001.zip › Supplemental Figures S1-S9.pdf]

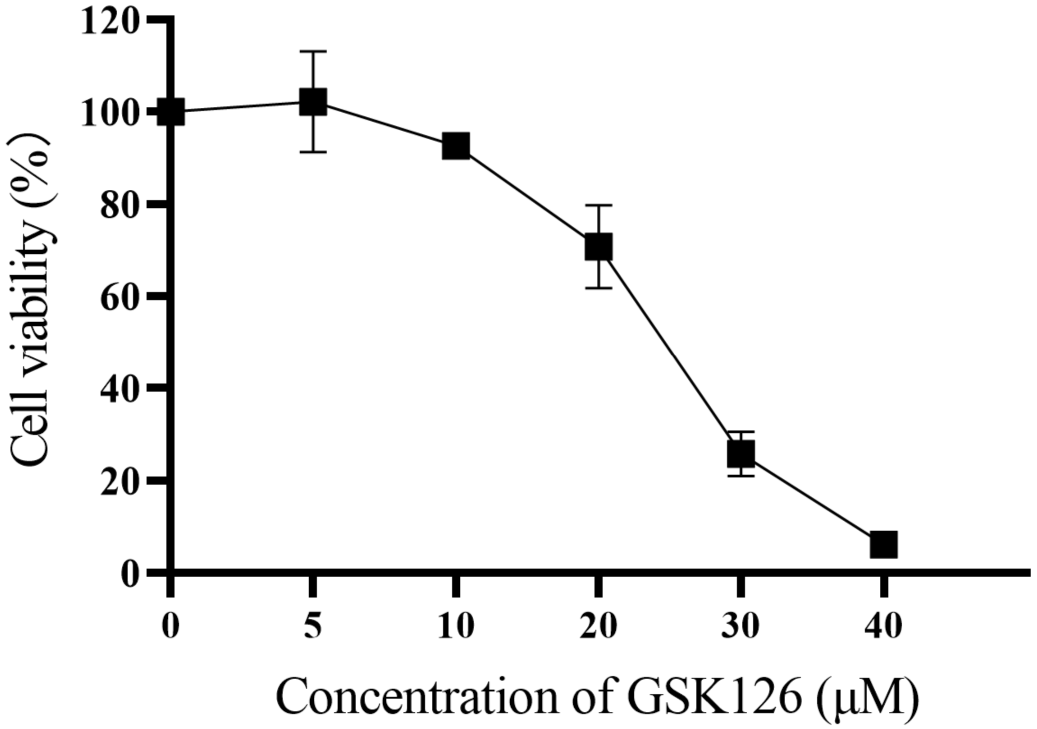

Figure S1 CCK-8 assay to select the concentration of GSK126 for the treatment of 3D4 cells

The viability of 3D4/21 AM cells treated with different concentrations of GSK126 (0, 5, 10, 20, 30 and 40 μM) for 24h. The results showed that the activity of 3D4/21 cells decreased gradually with the increase of GSK126 concentration. When the concentration of GSK126 was 5 μM, the activity of 3D4/21 cells decreased least. Therefore, 5 μM was chosen for subsequent experiments.

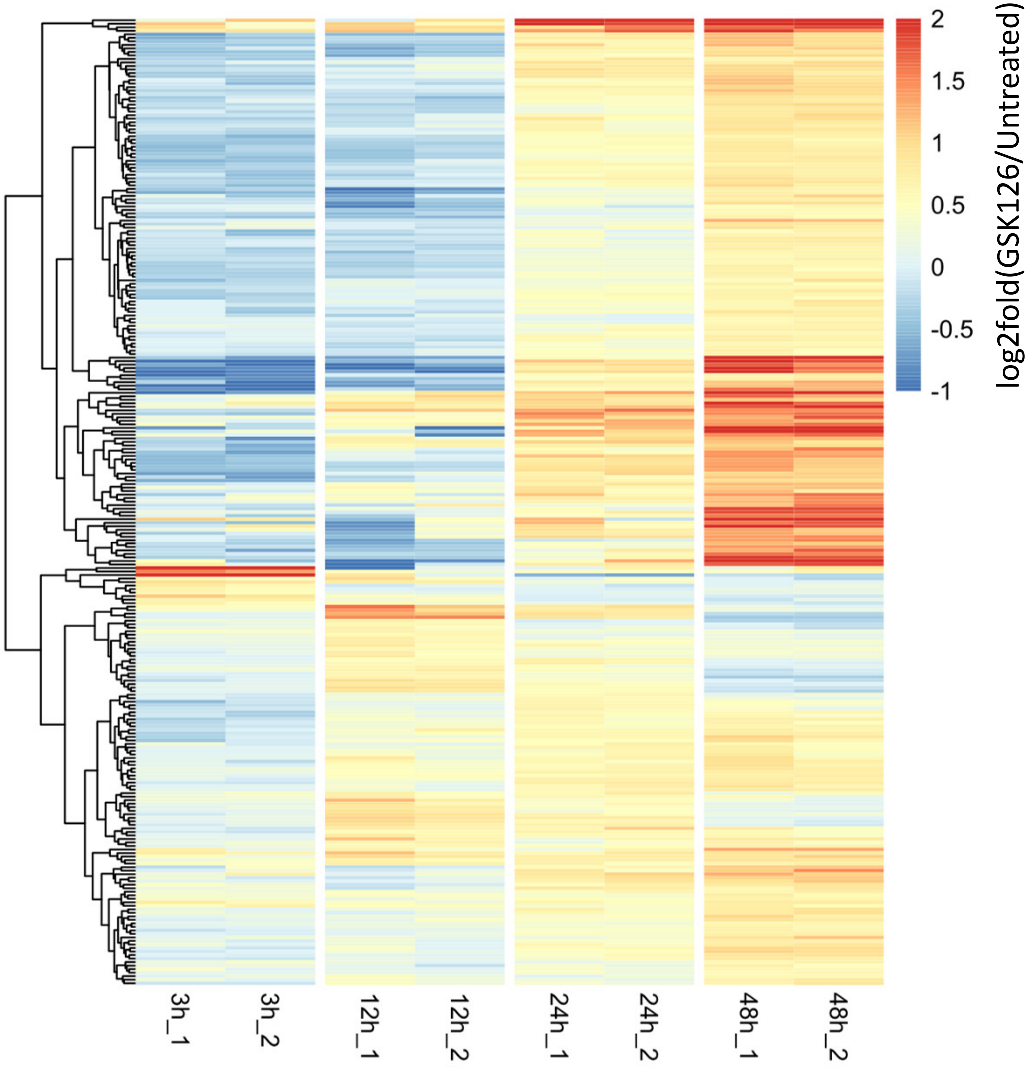

Figure S2 Expression changes of the significant DEGs after the inhibition of EZH2 in porcine 3D4/21 AMs

This heatmap shows the expression pattern of the up-regulated genes at different time periods (3h, 12h, 24h, 48h) after GSK126 treatment in porcine 3D4/21 AMs. This figure is related to Figure 1H.

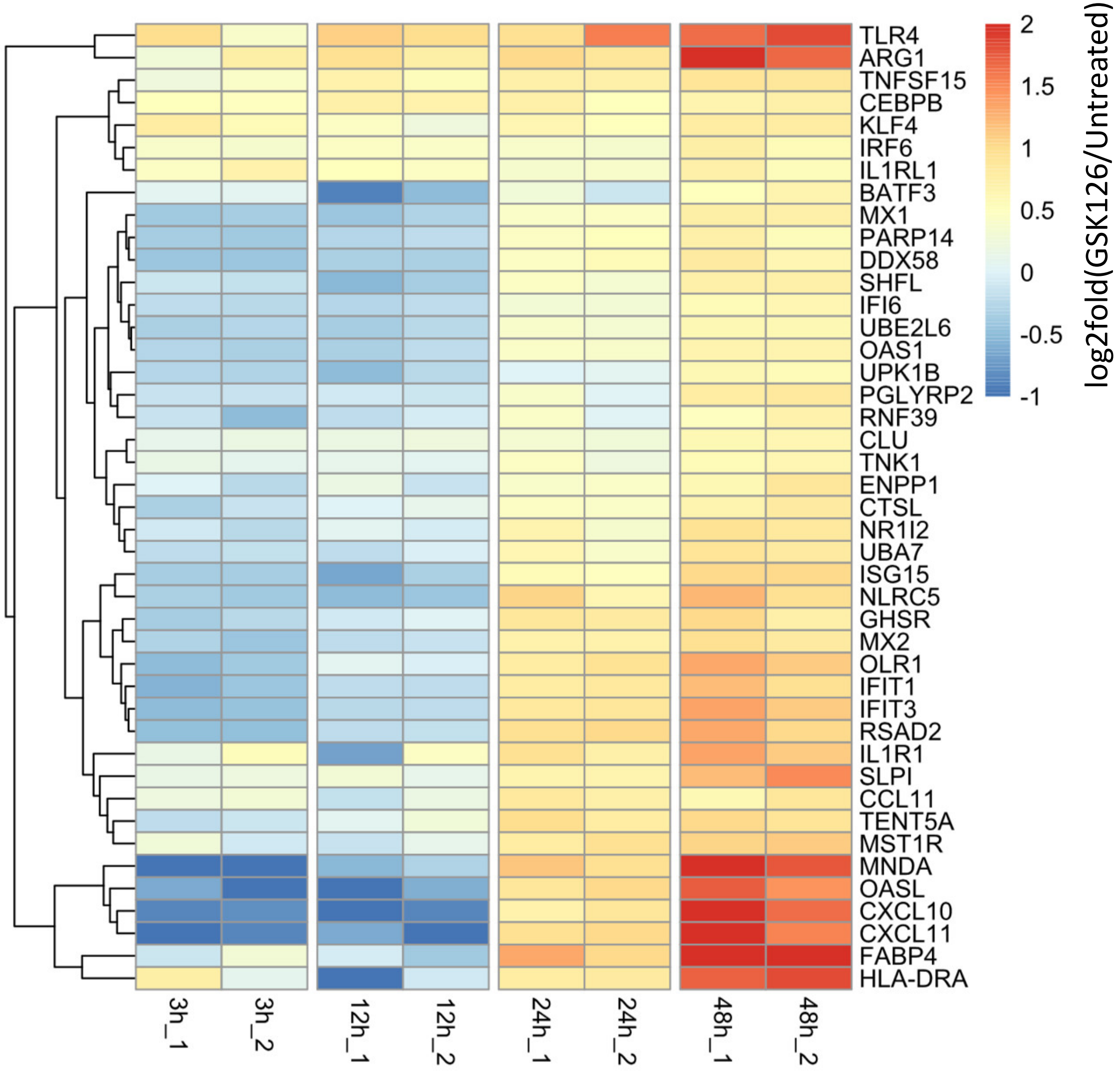

Figure S3 Expression changes of the immune-related DEGs after the inhibition of EZH2 in porcine 3D4/21 AMs

This heatmap shows the altered expression of the immune-related genes that are up-regulated after 48 hours of treatment in porcine 3D4/21 AMs. The immune-related genes were selected based on the GO enrichment analysis results. This figure is related to **Figure 2D**.

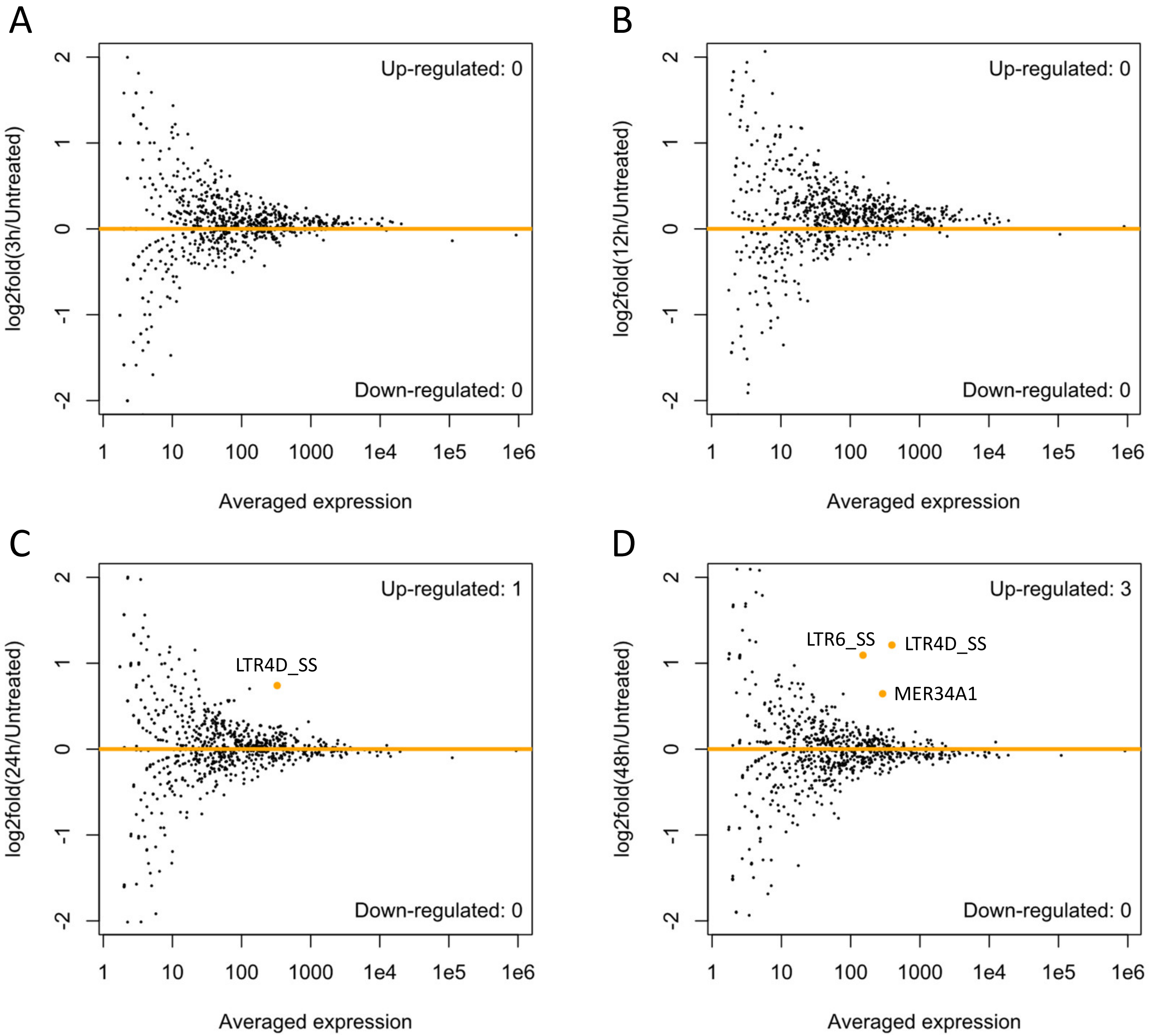

Figure S4 Differential expression of TE families after inhibition of EZH2 based on RNA-Seq data generated with PolyA-enrichment protocol

The MA plots show the differential expression of TE families in 3D4/21 AMs after treatment with the EZH2 inhibitor GSK126 for different time periods (3, 12, 24, 48 hours). The results are generated based on the analysis of the RNA-Seq datasets generated with PolyA-enrichment protocol.

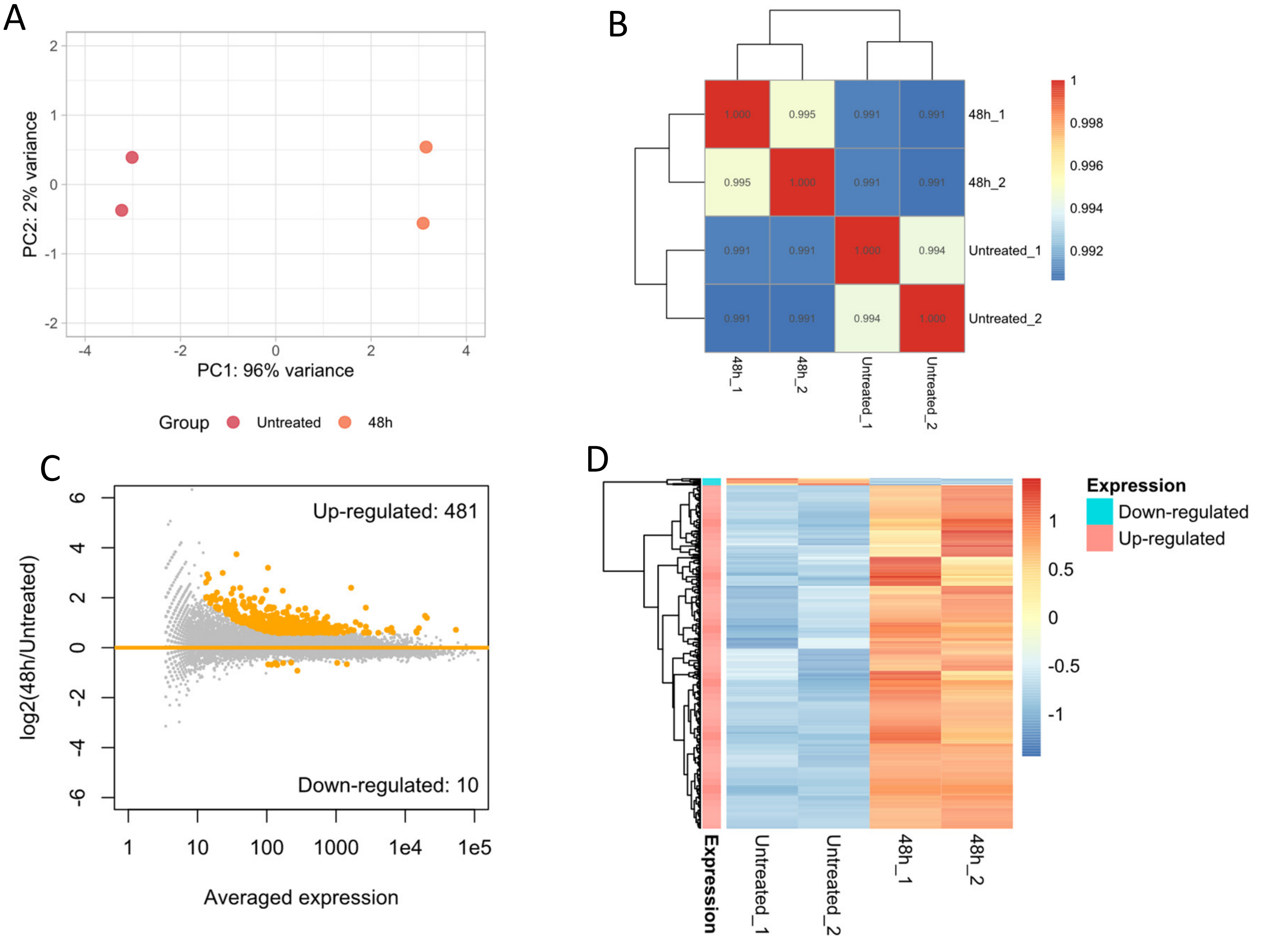

Figure S5 Altered gene expression after EZH2 inhibition in 3D4 cells based on rRNA-depleted RNA-Seq data
(**A**) PCA plot show the relationship between untreated and GSK126 treated (48 hours) 3D4 cells. (**B**) Heatmap shows the relationship between untreated and GSK126 treated (48 hours) 3D4 cells. The color gradient represents the Pearson's *r* among samples calculated based on the normalized read counts of all genes. (**C**) MA plot shows the significant gene expression in 3D4 cells due to EZH2 inhibition. (**D**) Expression profiles of the significant DEGs identified in 3D4 cells after EZH2 inhibition. The color gradient represents the row z-score.

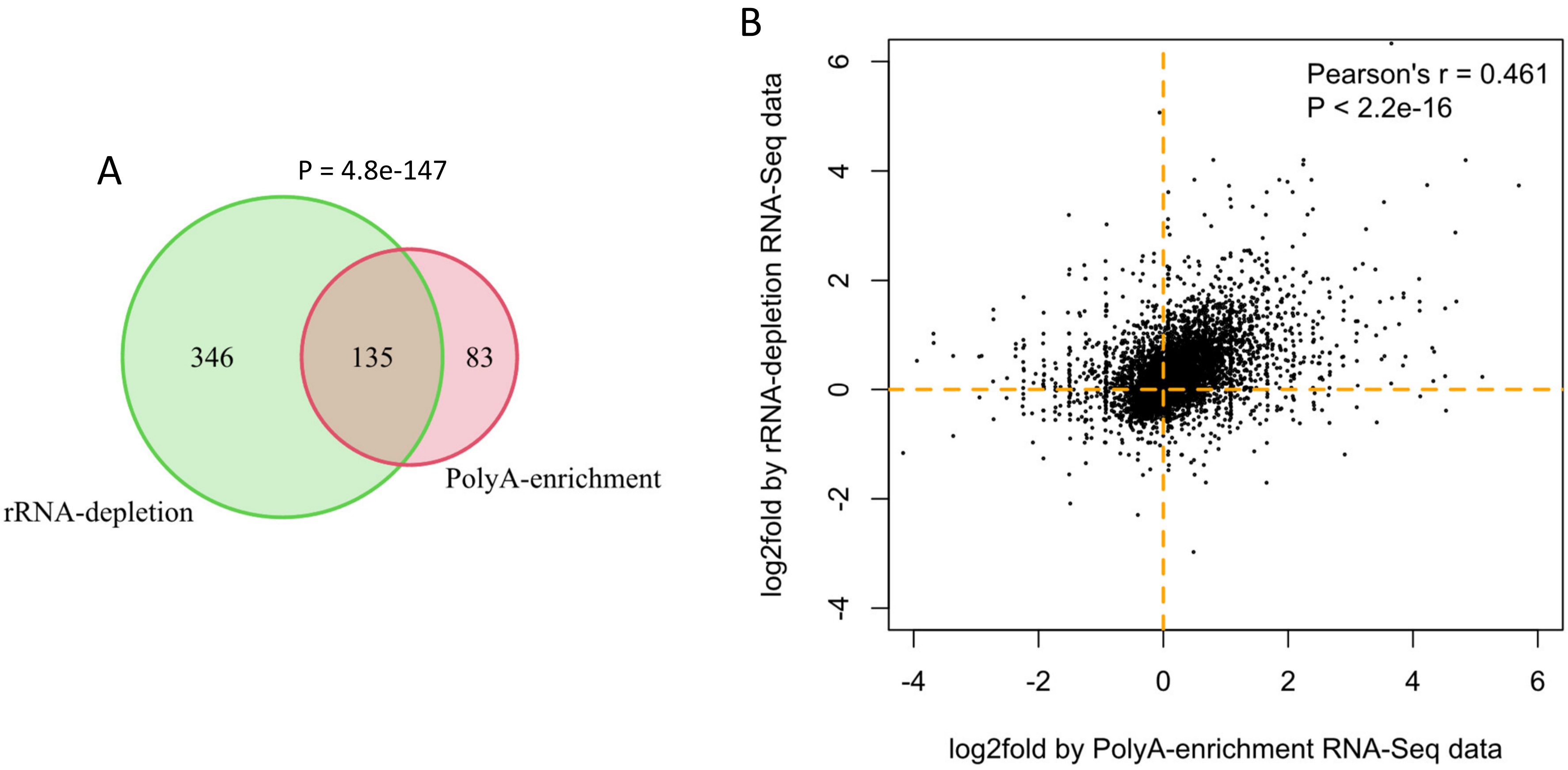

Figure S6 High consistency of the DEGs of untreated and GSK126 treated 3D4 cells identified based on the polyA-enrichment and rRNA-depletion protocols

(**A**) Venn diagram shows the overlap of the DEGs identified by using the RNA-Seq data generated by using the two different protocols. The P-value calculated by using Fisher's Exact Test is indicated. (**B**) Scatter plot shows the correlation of the log2fold expression changes calculated from the RNA-Seq data generated by using the two different protocols. Pearson's r and P-value are indicated.

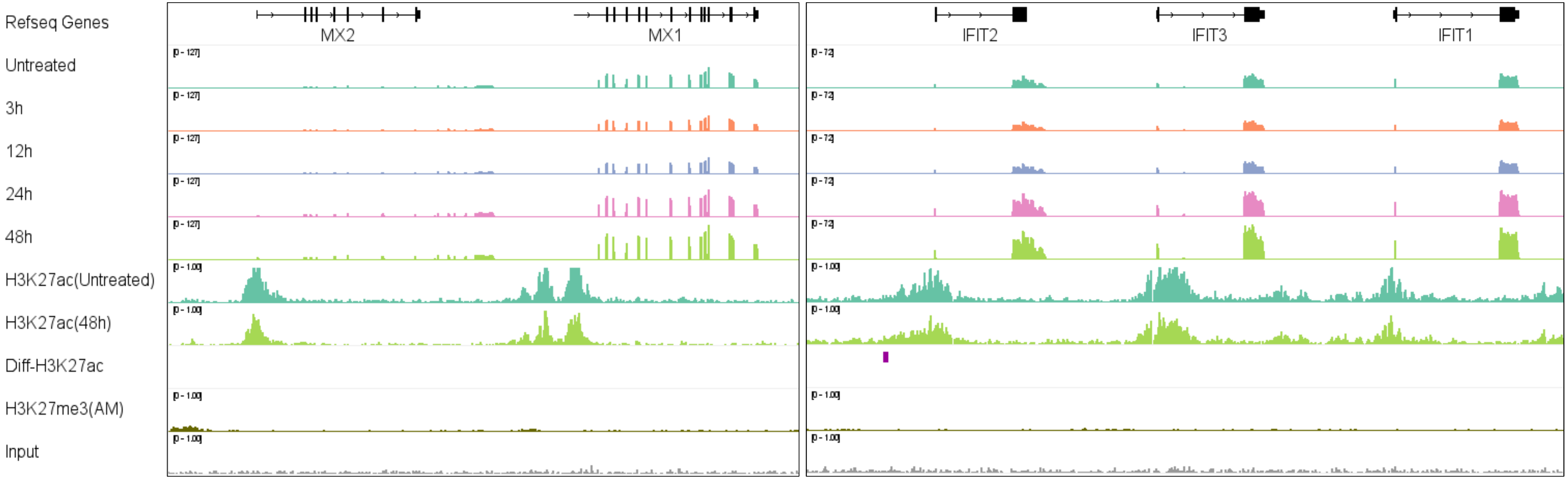

Figure S7 Demo tracks for the transcriptomic and epigenetic patterns surrounding MX1/2 and IFIT1/2/3 loci

The IGV tracks show the transcriptomic (RNA-Seq)and epigenomic (H3K27ac and H3K27me3) patterns surrounding MX1/2 and IFIT1/2/3 which are canonical ISGs. The 5 bars at top are based on RNA-Seq data after different time periods of GSK126 treatment.

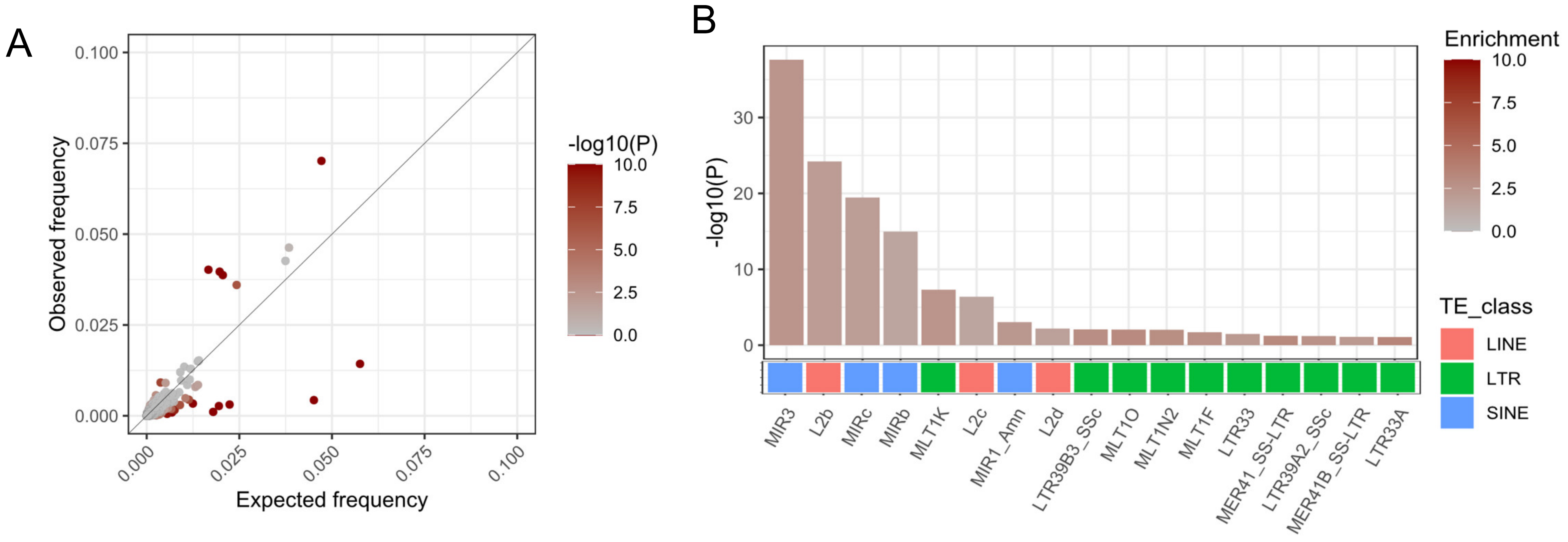

Figure S8 Identification of the TE families that are significantly overlap the loci with increased H3K27ac intensity after the inhibition of EZH2

(A) Scatter plot shows the TE enrichment analysis results. The color gradient indicates the –log10(P). (B) Barplot shows the details for the 17 TE families that are significantly enriched within the loci with increased H3K27ac intensity after the inhibition of EZH2 in porcine 3D4/21 AM cells.

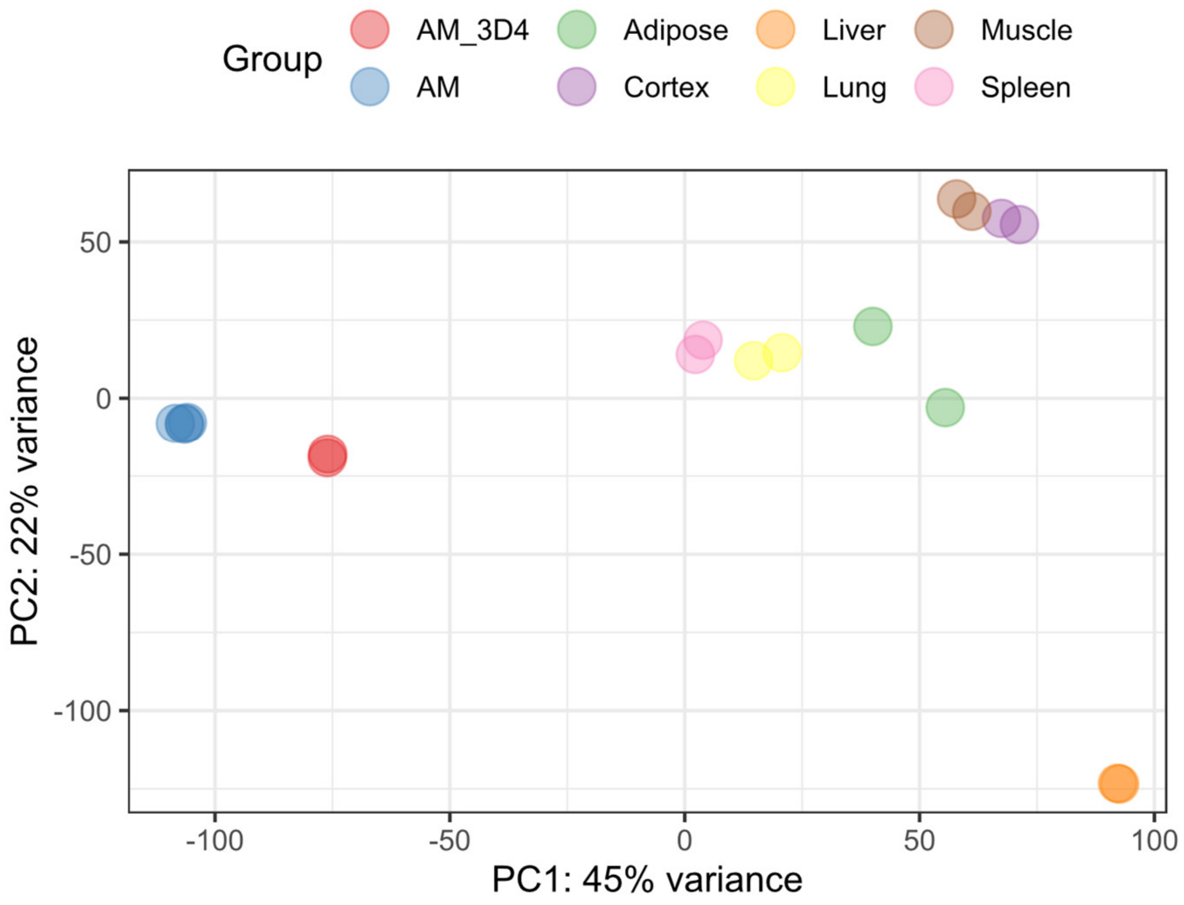

Figure S9 Relationship among porcine 3D4 AMs, primary AMs and various tissues based on transcriptomic data

This figure shows the PCA plot that visualized the relationship between porcine 3D4 AMs, primary AMs and various tissues (adipose, cortex, liver, lung, muscle and spleen). PCA analysis was performed on the normalized gene-level read counts calculated from corresponding RNA-Seq. Data for porcine 3D4 AMs were generated by this study. Data for primary porcine AMs were retrieved from Herrera-Uribe et al., Front Genet, 2020 (PMID: 32973863), and data for different porcine tissues were retrieved from Pan et al., Nature Comm. 2021 (PMID: 34615879).
