# Supplementary material for: Inhibition of EZH2 Causes Retrotransposon Derepression and Immune Activation in Porcine Lung Alveolar Macrophages
Source: Int J Mol Sci. 2023 Jan 25;24(3):2394. doi: 10.3390/ijms24032394 (PMC9917017; doi:10.3390/ijms24032394)
Supplement: Supplementary file 1 [file ijms-24-02394-s001.zip › Table S5.pdf]

Table S5 Primers used for the RT-PCR of IAV M genes

Gene name	Primer sequence (5'→3')	Sequence length (bp)
M	F: GTGCCGTCGGATGGTAGT<br>R: CAGTGATGAACCGCAGGAT	154
